# The best place to die: A reflection on dignity and context

**DOI:** 10.1017/S1478951525101338

**Published:** 2025-12-29

**Authors:** Bianca Sakamoto Ribeiro Paiva, Talita Caroline de Oliveira Valentino, Harvey Max Chochinov, Carlos Eduardo Paiva

**Affiliations:** 1 GPQual – Research Group on Palliative Care and Quality of Life – Barretos Cancer Hospital, Barretos, Brazil; 2 Teaching and Research Institute, Barretos Cancer Hospital, Barretos, Brazil; 3 Faculty Ceres (FACERES), São José do Rio Preto, Brazil; 4 Department of Psychiatry, Research Institute of Oncology and Hematology, Cancer Care Manitoba, Manitoba, Canada

Discussions, research, and reflections on the place of death have long been central to palliative care, where it is often considered a key indicator of quality. Dying at home is frequently regarded as the ‘gold standard’ of a good death (Tang and Bruera 2020; Wachterman et al. 2022), representing a key marker of quality at the end of life. This assumption is reinforced by healthcare professionals, care models, public policies, and healthcare systems, which often frame home death as the ideal scenario. However, growing evidence from research and clinical practice (Tang and Bruera 2020; Vidal et al. 2022; Wachterman et al. 2022) shows that the best place to die is not universal. It is not, by definition, home, hospital, hospice, or any other specific setting. Rather, it is the place that aligns with the patient’s values, needs, preferences, and circumstances, reflecting both personal and contextual factors. In other words, the question is less about ‘where’ and more about ‘how’ we allow people to die, with safety, meaning, and dignity.

The preference for dying at home has historically been associated with autonomy, dignity, and the comfort of being surrounded by loved ones in a familiar environment (Higginson et al. 2017; Pike et al. 2025). As such, this ideal is deeply embedded in care models, policy frameworks, and academic discourse within palliative care. However, this notion – sometimes romanticized – often fails to account for the structural challenges related to the home environment, including inadequate or unsafe physical conditions for complex care needs; logistical and social constraints such as lack of access to home-based palliative care or absence of trained family caregivers; and the emotional burden placed on families who may feel unprepared for caregiving responsibilities. These contextual realities significantly influence whether dying at home is truly a feasible or desirable option for each patient. For some, home represents love and belonging; for others, it represents fear, isolation, and the absence of professional support.

In a developing country like Brazil, characterized by social inequalities, cultural taboos around death, and limited healthcare access, the implementation of palliative care becomes especially challenging. The country ranks 42nd out of 80 in the Global Quality of Death Index, reflecting major gaps in the availability and quality of these services.

In a longitudinal study we recently published (Valentino et al. 2023), we observed that the way conversations were initiated about place-of-death preferences, and the factors influencing those preferences, was crucial to fostering meaningful dialogue and facilitating data collection. To support this process, we developed and validated a carefully worded introductory text designed to create a welcoming environment and help overcome initial conversational barriers:

*There is a well-known saying that the only certainty in life is that one day we will all die. This thought becomes even more tangible when someone is diagnosed with a life-limiting illness, such as cancer. Modern medicine constantly strives to prolong life and improve its quality. However, in some advanced cases, it must also focus on offering comfort and alleviating suffering for both patients and their families, always respecting the patient’s autonomy at every stage of life.*

Based on this premise, that death is an inevitable experience for all, participants were invited to reflect on which factors they considered most important when making decisions about their final moments. The survey questions included both structured, ranked response options and open-ended prompts. Participants were encouraged to consider aspects of their own lives, such as family support networks, housing conditions, socioeconomic status, and access to healthcare services. From this perspective, they were asked about their preferred place and conditions for end-of-life care should their health deteriorate significantly. Additionally, preferences were explored in four specific scenarios: (1) severe clinical deterioration without further specification, (2) clinical deterioration suffering from severe symptoms, (3) clinical deterioration receiving home-based visits, and (4) clinical deterioration receiving home-based visits and suffering from severe symptoms. Participants were also asked about places where they would not want to die under any circumstances.

It is important to emphasize that such preferences are not static; they are dynamic and may evolve over the course of illness. The place that seems ideal at the time of diagnosis may no longer align with the patient’s needs or wishes as death approaches. Decision-making, therefore, requires balancing multiple considerations, including the desire not to become a burden to family, the need for specialized symptom management, the pursuit of privacy, dignity, and spiritual peace, as well as emotional concerns such as the fear of loneliness or abandonment, regardless of the setting.

Recognizing the patient–family dyad as a central element in palliative care, it becomes clear that families navigate their own emotional challenges when considering their willingness and capacity to provide care at the end of life. Many families experience profound emotional exhaustion, guilt, and, at times, traumatic distress. Within this context, Chochinov’s model of dignity in the terminally ill (Chochinov et al. 2002) becomes especially relevant, emphasizing that dignity is not solely an internal experience but one that is co-constructed through relationships with others. Determining the best place to die, therefore, involves not only what matters to the patient but also what is sustainable and meaningful for those providing care. A dignified death, therefore, is relational; it exists in the space between someone who is dying and someone who is willing to stay.

Ensuring a dignified death cannot be reduced to predefined standards or assumptions about place. What truly matters is that, regardless of the setting, care is delivered in a way that fosters comfort, respect, love, and dignity. Rather than focusing on metrics such as ‘dying at home’, we must look at each person’s unique circumstances, needs, and preferences. Ultimately, the best place to die is the place where the person feels safe, supported, and where their dignity is preserved until the very end.
